# Supplementary material for: Phosphorus-activated carboxyl small molecule positive electrode for high specific capacity and long-life iron-organic batteries
Source: Nat Commun. 2026 Mar 15;17:4001. doi: 10.1038/s41467-026-70800-w (PMC13136356; doi:10.1038/s41467-026-70800-w)
Supplement: Supplementary file 1 — Supplementary Information [file 41467_2026_70800_MOESM1_ESM.pdf]

Supplementary Information

Phosphorus-Activated Carboxyl Small Molecule Positive Electrode for High Specific Capacity and Long-Life Iron-Organic Batteries

Yehui Zhang[1], Qi Huang[2], Pingxuan Liu[1], Yaokang Lv[3], Ziyang Song[1,4], Lihua Gan[1,5], Mingxian Liu[1,5]*

[1]Shanghai Key Lab of Chemical Assessment and Sustainability, School of Chemical Science and Engineering, Tongji University, 1239 Siping Rd., Shanghai, 200092, P. R. China.

[2]Institute for Electric Light Sources, School of Information Science and Technology, Fudan University, 2005 Songhu Rd., Shanghai, 200438, P. R. China.

[3]College of Chemical Engineering, Zhejiang University of Technology, 18 Chaowang Rd., Hangzhou, 310014, P. R. China.

[4]State Key Laboratory of Pollution Control and Resource Reuse, College of Environmental Science and Engineering, Advanced Research Institute, Tongji University, 1239 Siping Rd., Shanghai, 200092, P. R. China.

[5]State Key Laboratory of Cardiovascular Diseases and Medical Innovation Center, Shanghai East Hospital, School of Medicine, Tongji University, 150 Jimo Rd., Shanghai, 200120, P. R. China.

Section S1. Supplementary Characterizations

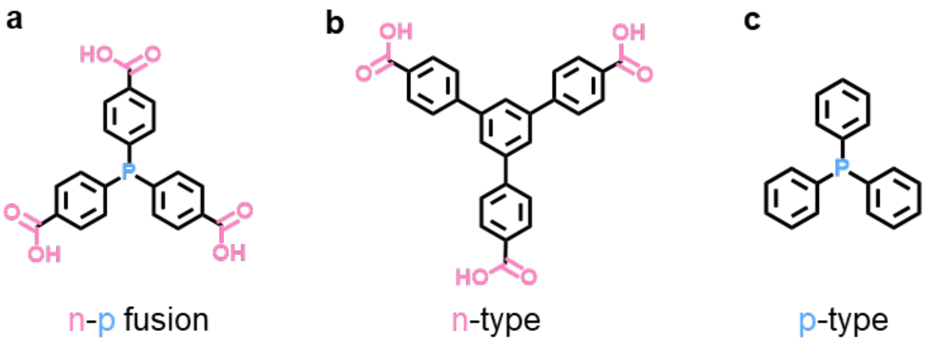

Supplementary Fig. 1 | Molecular structures. a bipolar PTBA, **b** n-type TCB and **c** p-type TPP.

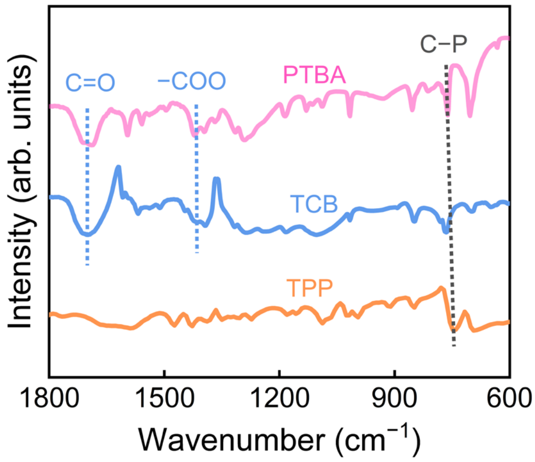

Supplementary Fig. 2 | FT-IR spectra of PTBA, TCB and TPP compounds.

Notes to Supplementary Fig. 2: FT-IR spectra exhibit the presence of C−P groups at 745 cm^{-1} in PTBA and TPP, and the asymmetric and symmetric stretching vibrations of C=O at 1685 cm^{-1} and −COO at 1410 cm^{-1} in PTBA and TCB. These results indicate the redox bipolarity of PTBA with dual −COOH and C−P active sites.

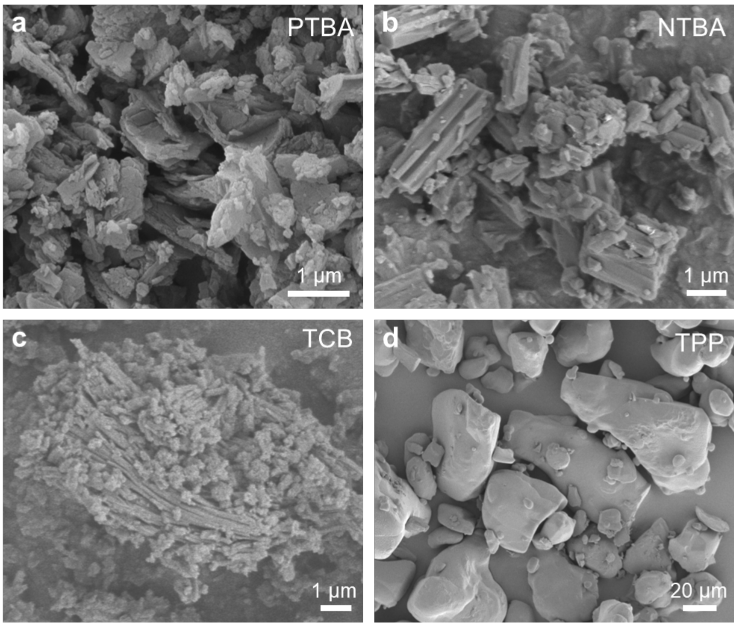

Supplementary Fig. 3 | SEM images. a PTBA, **b** NTBA, **c** TCB and **d** TPP.

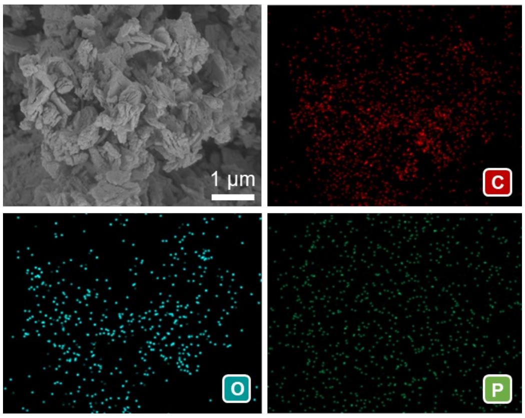

Supplementary Fig. 4 | Element distribution maps of PTBA.

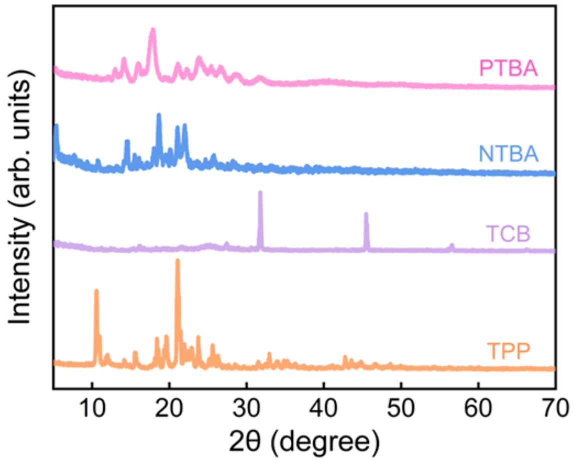

Supplementary Fig. 5 | XRD patterns of four organic molecules.

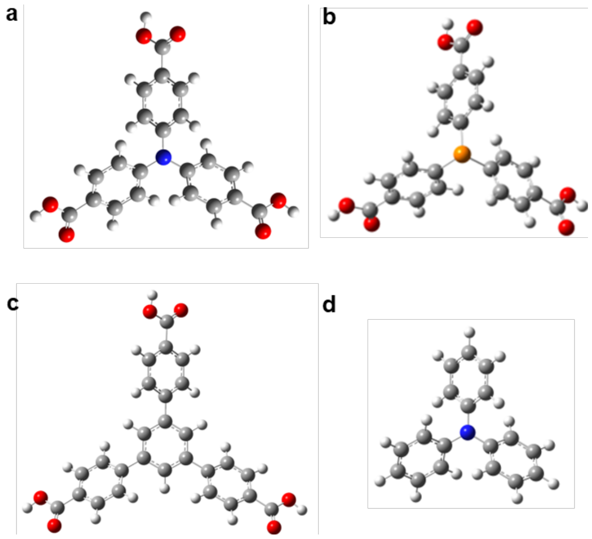

Supplementary Fig. 6 | Optimized molecular structures. **a** PTBA, **b** NTBA, **c** TCB and **d** TPP. Colours of elements: C, grey; O, red; H, white; P, blue; N, yellow.

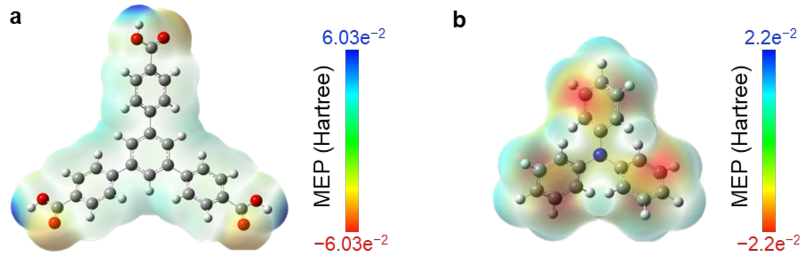

Supplementary Fig. 7 | MEP maps. **a** TCB and **b** TPP molecules. Colours of elements: C, grey; O, red; H, white; P, blue.

Notes to Supplementary Fig. 7: On the van der Waals surface of organics, carboxyl motifs of TCB show negative MEP values, which can coordinate with cations during discharging. C−P motif of TPP show positive MEP values, which tend to couple with anions during charging.

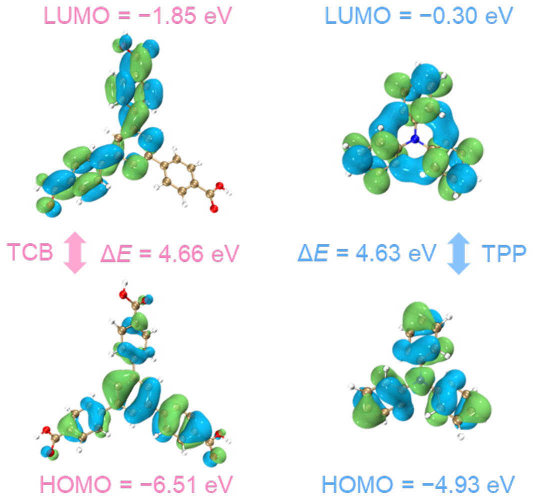

Supplementary Fig. 8 | LUMO/HOMO energy levels of TCB and TPP molecules. Blue regions: positive phase of the orbital wave function; Green regions: negative phase of the orbital wave function.

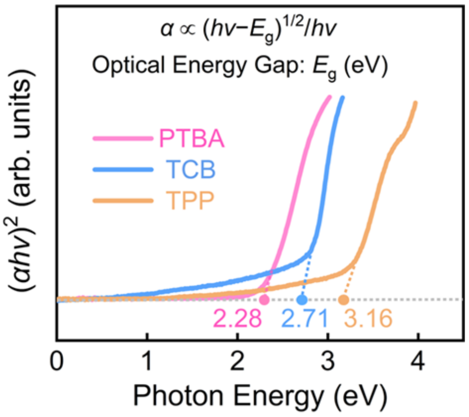

Supplementary Fig. 9 | Calculated E_g values of three organic materials.

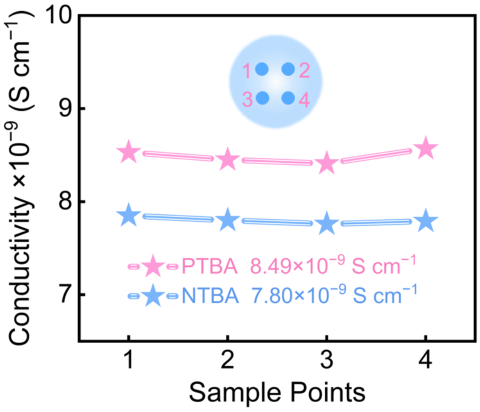

Supplementary Fig. 10 | Electrical conductivities tested at 4 different points on the thin sheets of PTBA and NTBA small molecules.

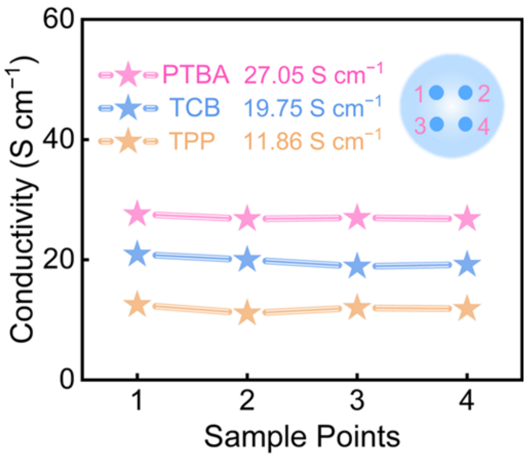

Supplementary Fig. 11 | Electrical conductivities tested at 4 different points on the three organic positive electrodes with an RTS-8 four-point probe.

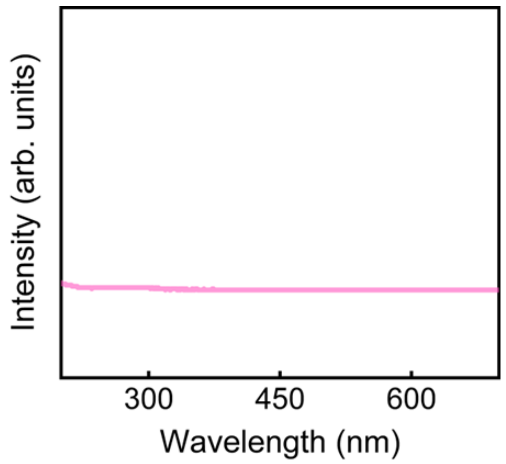

Supplementary Fig. 12 | UV-Vis spectrum of aqueous $Fe(OTF)_2$ electrolyte soaked with PTBA for three months.

Section S2. Supplementary Electrochemical Results

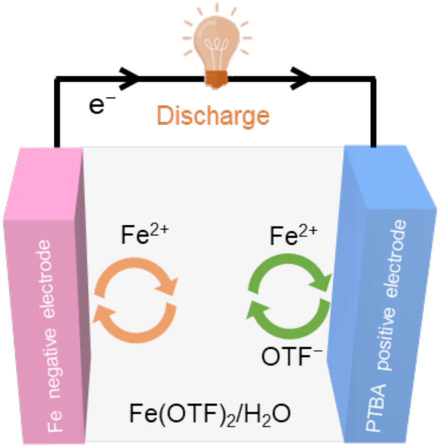

Supplementary Fig. 13 | Schematic configuration of Fe||PTBA cell, including Fe metal negative electrode, aqueous 1 M $Fe(OTF)_2$ electrolyte, and PTBA positive electrode.

Notes to Supplementary Fig. 13: Supplementary Fig. 13 was created using Microsoft PowerPoint, which is officially licensed to Tongji University (License ID: V2031185) for academic purposes, including research and publication.

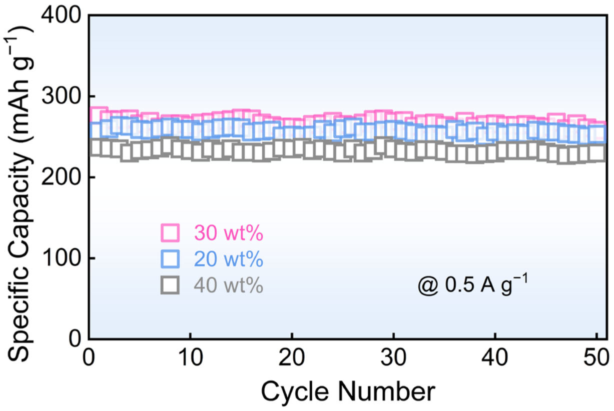

Supplementary Fig. 14 | The influence of acetylene black on discharge specific capacities of PTBA positive electrode.

Notes to Supplementary Fig. 14: Although increasing the content of conductive agent can improve the electrical conductivity of organic positive electrodes, it inevitably reduces the mass loading of active materials. This leads to a higher proportion of inactive components in the battery, thereby causing an unexpected reduction in specific capacity.[S1]

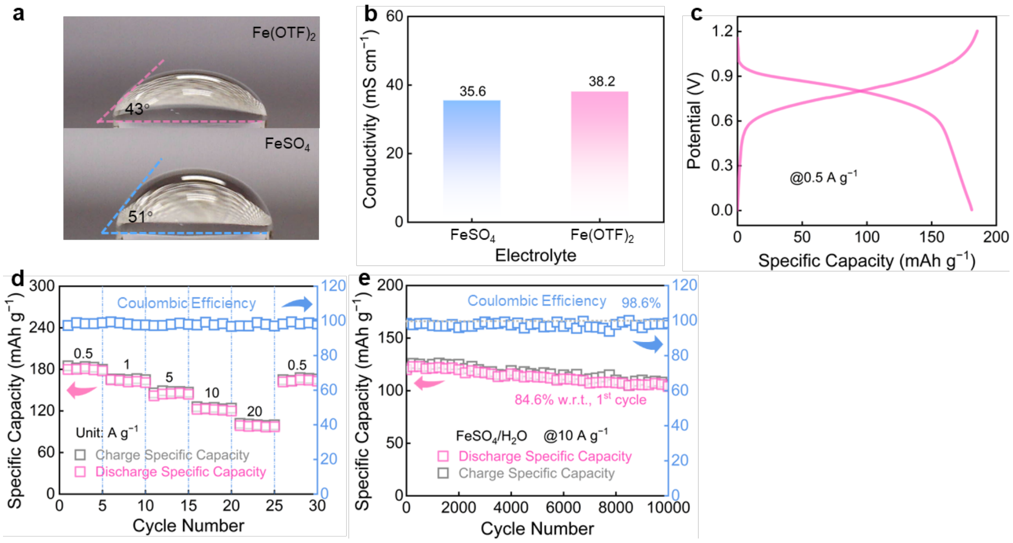

Supplementary Fig. 15 | Electrochemical performance characterization. a Contact angles and **b** ion conductivities of two different electrolytes. **c** A GCD curve, **d** rate capacities, **e** cycling stability of Fe||PTBA battery in 1 M $FeSO_4/H_2O$ electrolyte.

Notes to Supplementary Fig. 15: For a comparison, we studied the electrochemical properties of Fe||PTBA battery in 1 M $FeSO_4/H_2O$ electrolyte (Supplementary Fig. 15). Compared with $Fe(OTF)_2/H_2O$ electrolyte, $FeSO_4/H_2O$ electrolyte shows relatively low surface wettability (Supplementary Fig. 15a) and ionic conductivity (Supplementary Fig. 15b). As well-established, $CF_3SO_3^-$ ions demonstrate weaker solvation interactions with H_2O and metal ions than SO_4^{2-} anions, which promote efficient desolvation to liberate enhanced reaction kinetics and electrochemical activity (*Nat. Commun.* 2023, *14*, 3117).[S3] As a result, PTBA positive electrode in $FeSO_4/H_2O$ electrolyte delivers similar but slightly lower specific capacity, rate performance, and cycling stability (Supplementary Fig. 15c−e) than that of $Fe(OTF)_2/H_2O$ electrolyte. Such a result suggests the better adaptability of PTBA positive electrode in $Fe(OTF)_2/H_2O$ electrolyte, which thus is selected as the electrolyte.

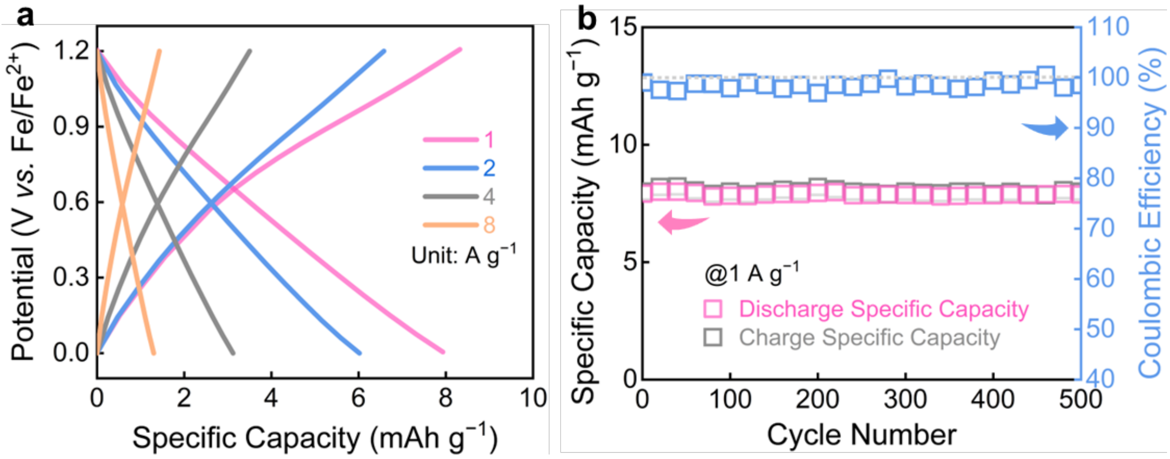

Supplementary Fig. 16 | Electrochemical performance characterization. a GCD profiles and **b** cycling performance of Fe||acetylene black battery in 1 M $Fe(OTF)_2/H_2O$ electrolyte.

<u>Notes to Supplementary Fig. 16</u>: The conductive agent of acetylene black shows an insignificant specific capacity contribution of 8 mAh g^{-1} at 1 A g^{-1}.

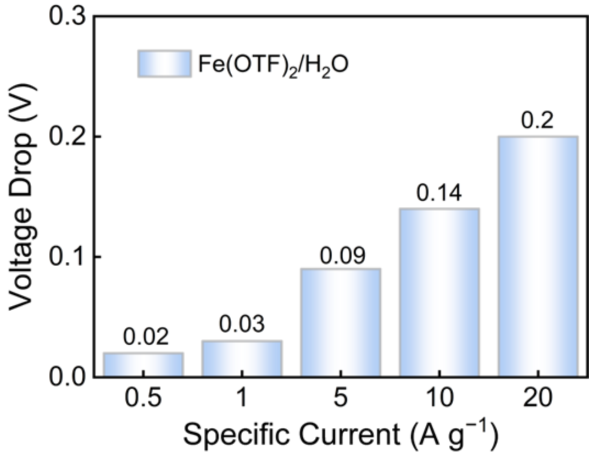

Supplementary Fig. 17 | Voltage drops of Fe||PTBA cell in discharge curves at various specific current.

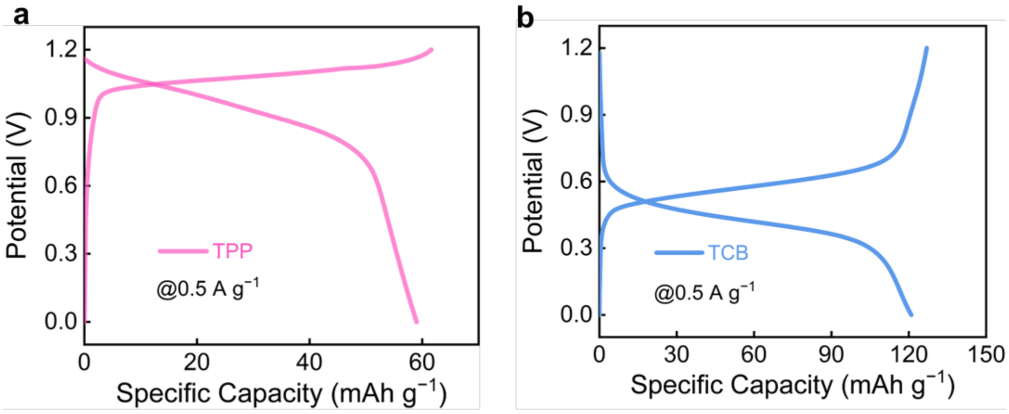

Supplementary Fig. 18 | GCD curves. a Fe||TPP and **b** Fe||TCB batteries in 1M Fe(OTF)$_2$/H_2O.

__**Notes to Supplementary Fig. 18**__**:** TCB (containging carboxyl) and TPP positive electrodes (containging phosphorus) exhibit relatively low specific capacity of 121 mAh g^{-1} (66% carboxyl utilization) and 59 mAh g^{-1} (57.7% phosphorus utilization, Supplementary Fig. 18), respectively, which can be further boosted to 276 mAh g^{-1} for PTBA positive electrode (containing carboxyl/phosphorus sites) with 98.5% utilization (Fig. 2b). This is because that PTBA positive electrode with P-extended conjugated structure liberates low energy gap (2.28 eV) compared with TCB/TPP positive electrodes (2.71/3.16 eV, Supplementary Fig. 9), which significantly increases the utilization of carboxyl redox sites. In addition, despite the 1 e$^-$ capacity contribution of P site (*vs.* total 4 e$^-$ charge storage), it boosts the average discharge voltage of PTBA to 0.8 V (Fig. 2b), higher than TCB positive electrode (0.5 V). These results highlight the importance of P group in PTBA for promoting high voltage and high carboxyl utilization for enhanced capacity storage.

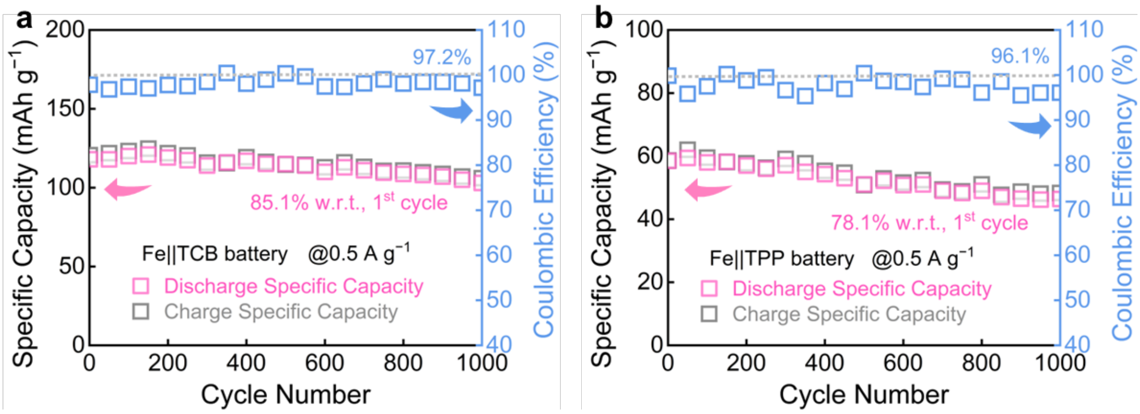

Supplementary Fig. 19 | Cycling stability. a Fe||TCB and **b** Fe||TPP batteries in 1M $Fe(OTF)_2/H_2O$.

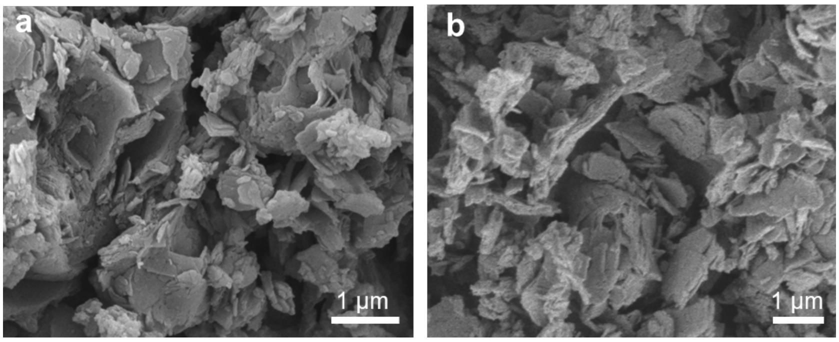

Supplementary Fig. 20 | SEM images of PTBA positive electrodes in $Fe(OTF)_2/H_2O$ electrolyte. a before and **b** after long-term cycling.

Notes to Supplementary Fig. 20: Of note, during the cycling process of Fe||PTBA battery, a thick glass fiber separator (675 μm) was used to alleviate the problem of inherent irreversibility and parasitic reactions in iron foil negative electrode (with a thickness of 50 μm) to achieve good cycle stability after 60,000 cycles. Such favorable electrochemical processes allow the high compatibility between PTBA positive electrode and Fe negative electrode for propelling Fe-organic batteries.

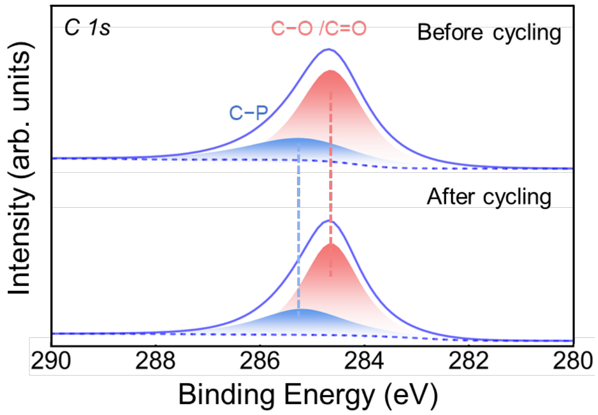

Supplementary Fig. 21 | *C 1s* XPS spectra of PTBA positive electrodes before and after prolonged cycling.

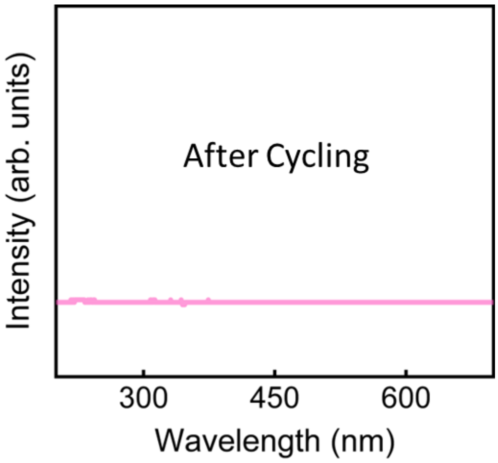

Supplementary Fig. 22 | UV-Vis spectrum of cycled PTBA positive electrode in $Fe(OTF)_2/H_2O$ electrolyte.

Notes to Supplementary Fig. 22: No UV/Vis absorption signals after cycling suggest the anti-dissolution effect of PTBA, which confirm the structural and functional robustness of PTBA positive electrode without causing capacity degradation.

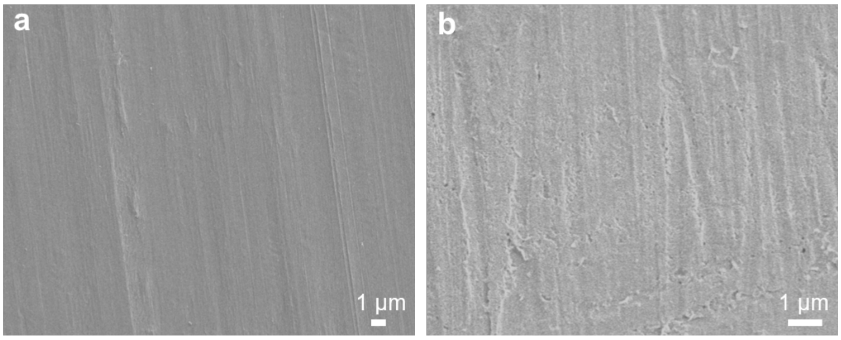

Supplementary Fig. 23 | SEM images of Fe negative electrodes in Fe||PTBA batteries. **a** before and **b** after long-term cycling.

Notes to Supplementary Fig. 23: Compared with pristine Fe foil (Supplementary Fig. 23a), the cycled Fe negative electrode shows rough surface geometries (Supplementary Fig. 23b), which derives from the repeat plating/stripping reaction of Fe negative electrode during long-term (dis)charged cycling processes. The structural degradation of Fe negative electrode is responsible for the capacity attenuation of Fe||PTBA battery after long-term cycling.

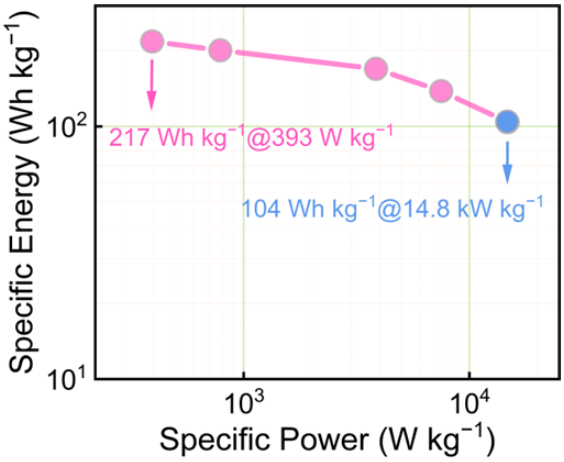

Supplementary Fig. 24 | Ragone plots of Fe||PTBA battery in 1 M Fe(OTF)$_2$/H$_2$O based on the mass loading of PTBA in the positive electrode (3 mg cm^{-2}).

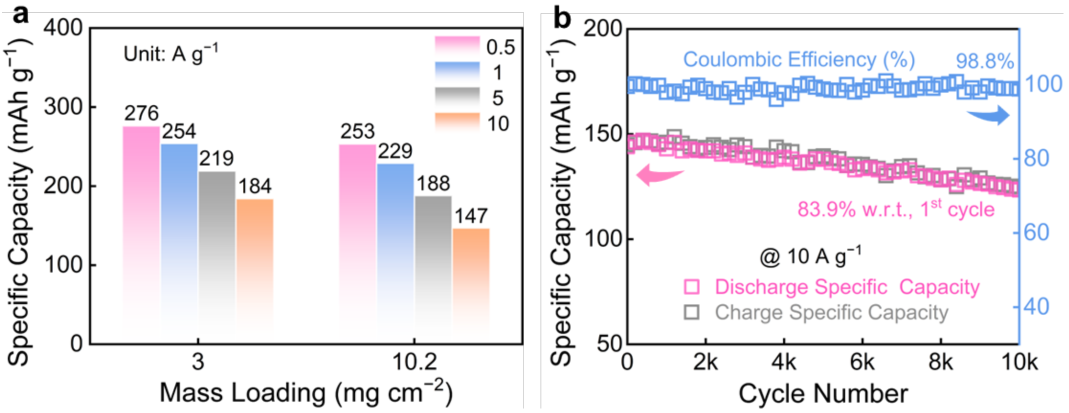

Supplementary Fig. 25 | Electrochemical performance of Fe||PTBA battery with a high-mass loading of 10.2 mg cm^{-2}$_{PTBA}$. a Rate metrics and **b** cycling stability.

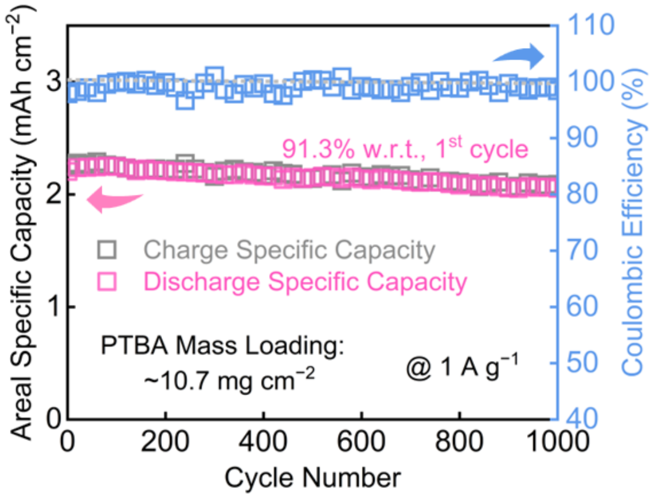

Supplementary Fig. 26 | Cycling stability of Fe||PTBA pouch cell at 1 A g^{-1}.

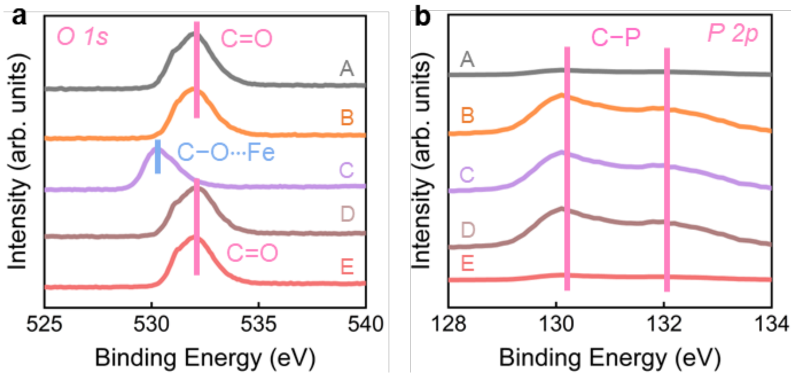

Supplementary Fig. 27 | XPS spectra of PTBA positive electrode corresponding to the selected voltages marked in the GCD curve. a *O 1s* and **b** *P 2p*.

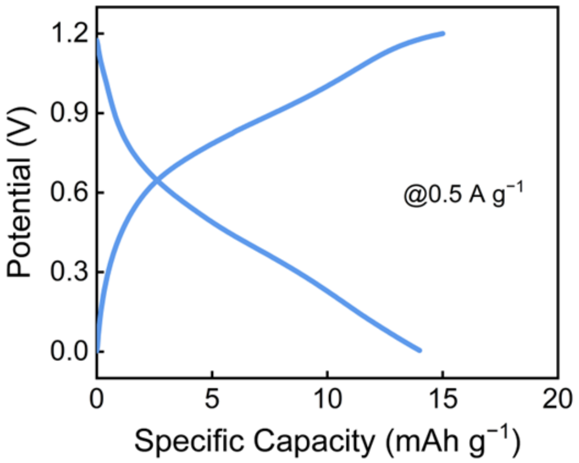

Supplementary Fig. 28 | A GCD curve of Fe||PTBA battery in HOTF/H_2O electrolyte (with the same pH value of $Fe(OTF)_2$/H_2O electrolyte).

Notes to Supplementary Fig. 28: Comparing with 1 M $Fe(OTF)_2$/H_2O electrolyte (pH=3~4), Fe||PTBA cell in HOTF/H_2O electrolyte displays completely different electrochemical behaviors with a negligible specific capacity contribution of 14 mAh g^{-1}, indicating that H^+ ions hardly participate in the electrochemical reaction process. Meanwhile, the slight acidity and low anion concentration of HOTF/H_2O electrolyte fail to effectively activate the redox activity of p-type C−P groups, leading to insignificant capacity storage. Thus, the role of H^+ in the operation of Fe||PTBA battery can be ignored.

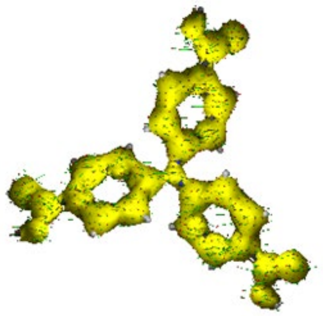

Supplementary Fig. 29 | The anisotropy of the induced current density (ACID) plots of PTBA.

Notes to Supplementary Fig. 29: ACID calculation shows that the diamagnetic continuous current flows over the backbone of PTBA, implying its high structure conjugation and π-aromaticity for supporting fast and stable electron transfer.

Section S3. Supplementary Table

Supplementary Table 1 | Comparison of specific capacity (C_m, mAh g^{-1}), specific energy (E, Wh kg^{-1}) and cycling life of recently reported organic materials for various metal batteries in the literatures.

Organic	Cell	C_m	E	Life	Refs.
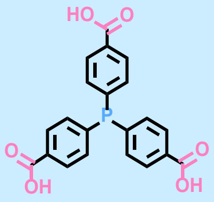 4,4',4"-Phosphanetriyltribenzoic Acid (PTBA)	Fe	276@0.5 A g^{-1}<br>141@20 A g^{-1}	217	77.6%, 60,000 cycles<br>10 A g^{-1}	This work
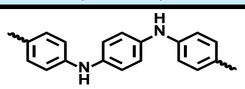 Polyaniline-sandwiched CNT (PANI-sandwiched CNT)	Fe	225@1.5 A g^{-1}<br>120@6 A g^{-1}	N/A	82%, 27000 cycles<br>4.5 A g^{-1}	[S2]
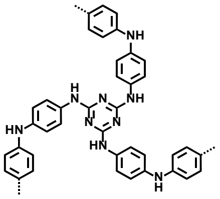 Cross-Linked Polyaniline (C-PANI)	Fe	209@5 A g^{-1}<br>133@25 A g^{-1}	N/A	84%, 39000 cycles<br>25 A g^{-1}	[S3]
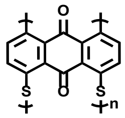 Anthraquinone-based Carbonyl Polymers (*d*-PAQS)	Mg	216@0.2 C<br>106@10 C	N/A	95%, 1000 cycles<br>10 C	[S4]
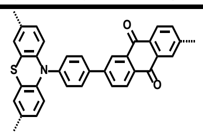 Donor-Acceptor Polymers (PPTZ-AQ)	Al	205@0.05 A g^{-1}<br>165@2 A g^{-1}	N/A	78.4%, 60000 cycles<br>5 A g^{-1}	[S5]
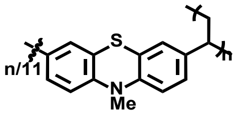 Poly(3-vinyl-N-methylphenothiazine) (X-PVMPT)	Al	167@0.5 C<br>64@100 C	355	88%, 5000 cycles<br>10 C	[S6]

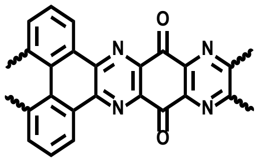<br>Poly(4,5-dihydrophenanthro[4,5-abc]pyrazino[2,3-i]phenazine-10,15-dione)<br>(PYTQ-CNT)	Al	295@0.1 A g^{-1}<br>85@2 A g^{-1}	413	74.6%, 4000 cycles<br>1 A g^{-1}	[S7]
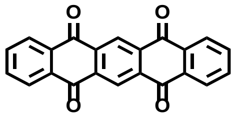<br>5,7,12,14-Pentacenetetrone<br>(PT)	Ca	150.5@5 A g^{-1}<br>86.1@100 A g^{-1}	200.4	114%, 3000 cycles<br>30 A g^{-1}	[S8]
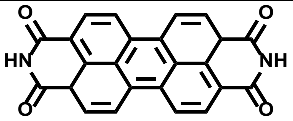<br>3,4,9,10-Perylenetetracarboxylic Diimide<br>(PTCDI)	Ca	131.8@0.1 A g^{-1}<br>86.2@10 A g^{-1}	N/A	72.7%, 68000 cycles<br>1 A g^{-1}	[S9]
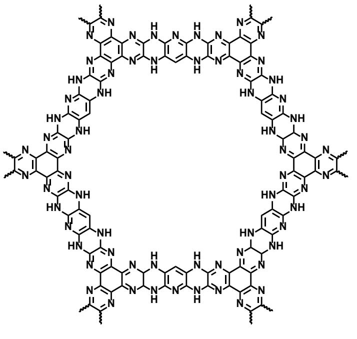<br>(PTHAT-COF)	Ca	155.5@1 A g^{-1}<br>60@20 A g^{-1}	159.7	83.6%, 10000 cycles<br>10 A g^{-1}	[S10]
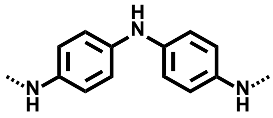<br>Polyaniline<br>(PANI)	Sn	0.3@2 mA cm^{-2}<br>0.15@20 mA cm^{-2}	N/A	82.7%, 1600 cycles<br>20 mA cm^{-2}	[S11]
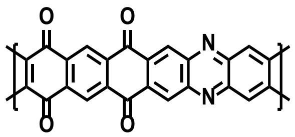<br>Poly(phenazine-alt-pyromellitic anhydride)<br>(PPPA)	Zn	210@0.05 A g^{-1}<br>140@5 A g^{-1}	N/A	70.6%, 20,000 cycles<br>5 A g^{-1}	[S12]

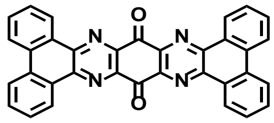 Dibenzo[a,c]dibenzo[5,6:7,8] quinoxalino[2,3-i]phenazine-10,21-dione (TABQ-PQ)	Zn	193@0.1 A g^{-1}<br>140.7@20 A g^{-1}	N/A	90.8%, 30,000 cycles<br>5 A g^{-1}	[S13]
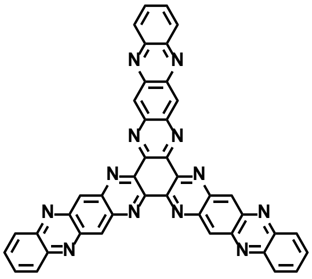 Hexaaz-atrinaphthalene-phenazine (HATN-PNZ)	Zn	225@5 A g^{-1}<br>131@60 A g^{-1}	153.9	92.7%, 30,000 cycles<br>30 A g^{-1}	[S14]
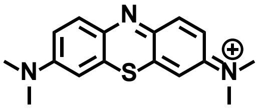 Methylene Blue (MB)	Zn	143@0.167 A g^{-1}<br>58@83.5 A g^{-1}	N/A	86%, 20,000 cycles<br>16.7 A g^{-1}	[S15]
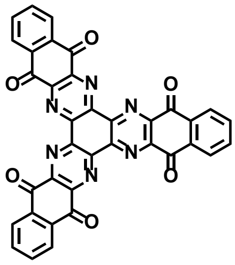 Hexaazatrinaphthalene (BQPH)	Zn	429@0.1 A g^{-1}<br>145@20 A g^{-1}	N/A	82%, 1000 cycles<br>10 A g^{-1}	[S16]
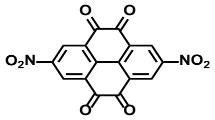 2,7-Dinitropyrene-4,5,9,10-Tetraone (DNPT)	Zn	320@0.2 A g^{-1}<br>113@50 A g^{-1}	226	81.2%, 60,000 cycles<br>10 A g^{-1}	[S17]
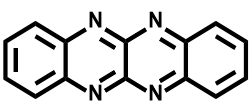 5,6,11,12-Tetraazanaphthacene (TANC)	Zn	213@0.1 C<br>120@20 C	245	71%, 47,500 cycles<br>10 C	[S18]

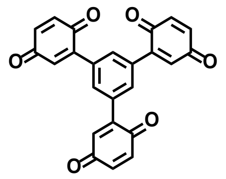<br>Phenyl Cross-Linked Tri-p-Benzoquinone (Ph-tri-BQ)	Zn	402@0.2 A g^{-1}<br>221@100 A g^{-1}	354	55%, 150,000 cycles<br>100 A g^{-1}	[S19]
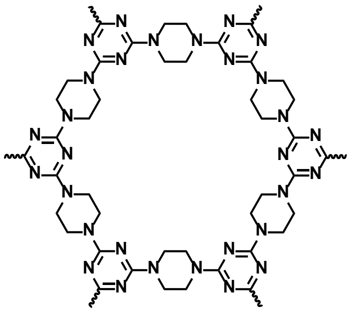<br>Amphoteric Organic Superstructures (AOSs)	Zn	465@0.1 A g^{-1}<br>151@50 A g^{-1}	412	80.5%, 70,000 cycles<br>10 A g^{-1}	[S20]
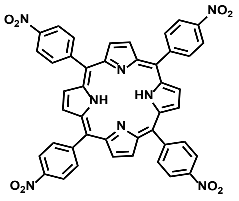<br>Tetranitroporphyrin (TNP)	Zn	338@0.2 A g^{-1}<br>155@20 A g^{-1}	365	71.6%, 50,000 cycles<br>10 A g^{-1}	[S21]
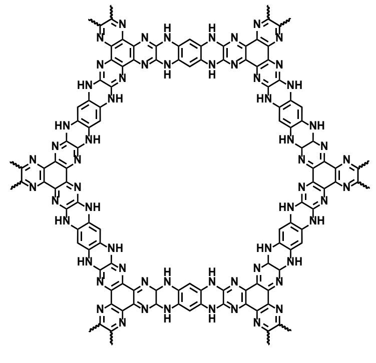<br>Covalent Organic Framework (HPP-COF)	Zn	184.3@5 A g^{-1}<br>91.7@100 A g^{-1}	N/A	104%, 40,000 cycles<br>30 A g^{-1}	[S22]
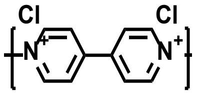<br>Viologen(bipyridine)-based Organic Framework (VOF)	Zn	113@0.25 A g^{-1}<br>82@20 A g^{-1}	68	70%, 45,000 cycles<br>5 A g^{-1}	[S23]

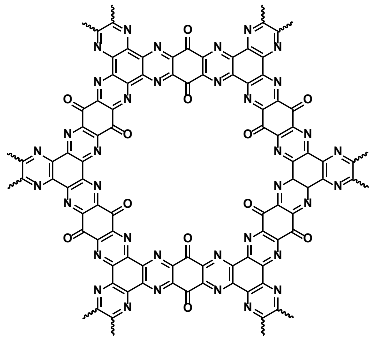 1,4,5,8,9,12-Hexaazatriphenylene-based COF (HAQ-COF)	Zn	344@0.1 A g^{-1}<br>95.6@10 A g^{-1}	N/A	88%, 10,000 cycles<br>5 A g^{-1}	[S24]
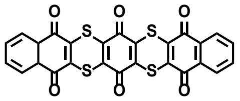 Benzo[b]naphtho[2’,3’:5,6][1,4]dithiino[2,3-i]thianthrene-5,7,9,14,16,18-hexone (BNDTH)	Zn	296@0.05 A g^{-1}<br>120@10 A g^{-1}	240	65%, 58,000 cycles<br>10 A g^{-1}	[S25]
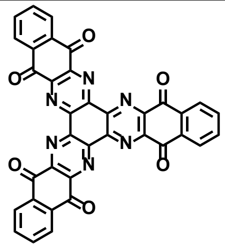 Hexaazatrinaphthalene-quione (HATNQ)	Zn	482.5@0.2 A g^{-1}<br>177.5@9 A g^{-1}	289	75%, 11,000 cycles<br>5 A g^{-1}	[S26]
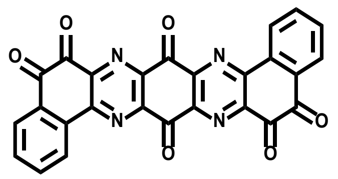 Benzo[a]benzo[7,8]quinoxalino[2,3-i]phenazine-5,6,8,14,15,17-hexane (BBQPH)	Zn	498.6@0.2 A g^{-1}<br>393.6@8 A g^{-1}	355	95%, 1000 cycles<br>5 A g^{-1}	[S27]
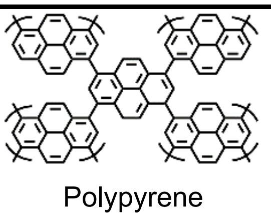 Polypyrene (CLPy)	Zn	180@0.05 A g^{-1}<br>105@3 A g^{-1}	N/A	96.4%, 38,000 cycles<br>3 A g^{-1}	[S28]

Supplementary Table 2 | Comparison of R_{ct} (Ω) parameter of PTBA positive electrode.

Temperature(°C)	R_{ct1} (Ω)	R_{ct2} (Ω)	R_{ct3} (Ω)	numerical errors
10	41.2	42	42.8	1.9%
20	29.2	28.8	28.9	0.7%
30	22.3	21.5	22.2	2.2%
40	17.3	17.1	16.3	3.5%
50	12.2	12.5	12.7	2.4%

Supplementary Table 3 | Comparison of R_{ct} (Ω) parameter of NTBA positive electrode.

Temperature(°C)	R_{ct1} (Ω)	R_{ct2} (Ω)	R_{ct3} (Ω)	numerical errors
10	73.7	74.2	77.1	2.8%
20	51.8	51.3	49.9	2.1%
30	36.3	33.8	35	3.4%
40	23.8	22.6	24.1	3.8%
50	16.5	17.1	17.6	2.9%

Section S4. Supplementary References

[S1] X. Huang, X. Qiu, W. Wang, J. Li, Z. Li, X. Yu, J. Ma, Y. Wang, *J. Am. Chem. Soc.* **2023**, *145*, 25604−25613.

[S2] Y. Zhang, C. Lee, M. Islam, J. Kwon, C. Yu, *Energy Environ. Sci.* **2025**,*18*, 1428-1439.

[S3] H. Lv, Z. Wei, C. Han, X. Yang, Z. Tang, Y. Zhang, C. Zhi, H. Li, *Nat. Commun.* **2023**, *14*, 3117.

[S4] X. Ren, D. Tao, S. Cui, T. Li, Y. Cao, F. Xu, *Energy Storage Mater.* **2023**, *63*, 102992.

[S5] L. Luo, C. Zhang, W. Ma, C. Han, X. Ai, Y. Chen, Y. Xu, X. Ji, J. Jiang, *Adv. Mater.* **2024**, *36*, 2406106.

[S6] G. Studer, A. Schmidt, J. Büttner, M. Schmidt, A. Fischer, I. Krossing, B. Esser, *Energy Environ. Sci.* **2023**, *16*, 3760.

[S7] X. Peng, Y. Xie, A. Baktash, J. Tang, T. Lin, X. Huang, Y. Hu, Z. Jia, D. J. Searles, Y. Yamauchi, L. Wang, B. Luo, *Angew. Chem. Int. Ed.* **2022**, *61*, e202203646.

[S8] C. Han, H. Li, Y. Li, J. Zhu, C. Zhi, *Nat. Commun.* **2021**, *12*, 2400.

[S9] F. Qiao, J. Wang, R. Yu, M. Huang, L. Zhang, W. Yang, H. Wang, J. Wu, L. Zhang, Y. Jiang, Q. An, *ACS Nano* **2023**, *17*, 23046.

[S10] C. Wang, R. Li, Y. Zhu, Y. Wang, Y. Lin, L. Zhong, H. Chen, Z. Tang, H. Li, F. Liu, C. Zhi, H. Lv, *Adv. Energy Mater.* **2023**, *13*, 2302495.

[S11] H. Zhang, Diyu Xu, Fan Yang, Jinhao Xie, Qiyu Liu, Di-Jia Liu, Minghao Zhang, Xihong Lu, Ying Shirley Meng, *Joule* **2023**, *7*, 971.

[S12] F. Ye, Q. Liu, H. Dong, K. Guan, Z. Chen, N. Ju, L. Hu, *Angew. Chem. Int. Ed.* **2022**, *61*, e202214244.

[S13] T. Sun, W. Zhang, Z. Zha, M. Cheng, D. Li, Z. Tao, *Energy Storage Mater.* **2023**, *59*, 102778.

[S14] S. Li, J. Shang, M. Li, M. Xu, F. Zeng, H. Yin, Y. Tang, C. Han, H. Cheng, *Adv. Mater.* **2022**, *34*, 2207115.

[S15] M. Tang, Q. Zhu, P. Hu, L. Jiang, R. Liu, J. Wang, L. Cheng, X. Zhang, W. Chen, H. Wang, *Adv. Funct. Mater.* **2021**, *31*, 2102011.

[S16] Z. Tie, Y. Zhang, J. Zhu, S. Bi, Z. Niu, *J. Am. Chem. Soc.* **2022**, *144*, 10301−10308.

[S17] Z. Song, L. Miao, Y. Lv, L. Gan, M. Liu, *Angew. Chem. Int. Ed.* **2023**, *62*, e202309446.

[S18] D. Du, J. Zhou, Z. Yin, G. Feng, W. Ji, H. Huang, S. Pang, *Adv. Energy Mater.* **2024**, *14*, 2400580.

[S19] L. Lin, Z. Xue, T. Qiu, J. Zhu, G. Zhang, H. Zhan, K. Wang, X. Sun, *Energy Environ. Sci.* **2024**, *17*, 6499.

[S20] Z. Song, Q. Huang, Y. Lv, L. Gan, M. Liu, *Angew. Chem. Int. Ed.* **2025**, *64*, e202418237.

[S21] Z. Song, L. Miao, H. Duan, Y. Lv, L. Gan, M. Liu, *Angew. Chem. Int. Ed.* **2024**, *63*, e202401049.

[S22] Y. Lin, H. Cui, C. Liu, R. Li, S. Wang, G. Qu, Z. Wei, Y. Yang, Y. Wang, Z. Tang, H. Li, H. Zhang, C. Zhi, H. Lv, *Angew. Chem. Int. Ed.* **2023**, *62*, e202218745.

[S23] L. Xie, K. Xu, W. Sun, Y. Fan, J. Zhang, Y. Zhang, H. Zhang, J. Chen, Y. Shen, F. Fu, H. Kong, G. Wu, J. Wu, L. Chen, H. Chen, *Angew. Chem. Int. Ed.* **2023**, *62*, e202300372.

[S24] W. Wang, V. S. Kale, Z. Cao, Y. Lei, S. Kandambeth, G. Zou, Y. Zhu, E. Abouhamad, O. Shekhah, L. Cavallo, M. Eddaoudi, H. N. Alshareef, *Adv. Mater.* **2021**, *33*, 2103617.

[S25] Q. Sun, T. Sun, J. Du, K. Li, H. Xie, G. Huang, X. Zhang, *Adv. Mater.* **2023**, *35*, 202301088.

[S26] Y. Chen, J. Li, Q. Zhu, K. Fan, Y. Cao, G. Zhang, C. Zhang, Y. Gao, J. Zou, T. Zhai, C. Wang, *Angew. Chem. Int. Ed.* **2022**, *61*, e202116289.

[S27] W. Li, H. Xu, H. Zhang, F. Wei, L. Huang, S. Ke, J. Fu, C. Jing, J. Cheng, S. Liu, *Nat. Commun.* **2023**, *14*, 5235.

[S28] C. Zhang, W. Ma, C. Han, L.-W. Luo, A. Daniyar, S. Xiang, X. Wu, X. Ji, J.-X. Jiang, *Energy Environ. Sci.* **2021**, *14*, 462.
