# Supplementary material for: Phosphorus-activated carboxyl small molecule positive electrode for high specific capacity and long-life iron-organic batteries
Source: Nat Commun. 2026 Mar 15;17:4001. doi: 10.1038/s41467-026-70800-w (PMC13136356; doi:10.1038/s41467-026-70800-w)
Supplement: Supplementary file 2 — Description of Additional Supplementary Files [file 41467_2026_70800_MOESM2_ESM.pdf]

Description of Additional Supplementary Files

Supplementary Data 1. Monomer: Optimized molecular geometries of PTBA, NTBA, TCB and TPP.

Supplementary Data 2. RDG: Optimized geometric models of two molecules for RDG analysis.

Supplementary Data 3. Coordinated Positive Electrode: Geometrically optimized structures of PTBA with OTF^- and Fe^{2+} coordination modes.
